# Parameter Values for Epidemiological Models of Foot-and-Mouth Disease in Swine

**DOI:** 10.3389/fvets.2016.00044

**Published:** 2016-06-01

**Authors:** Amy C. Kinsley, Gilbert Patterson, Kimberly L. VanderWaal, Meggan E. Craft, Andres M. Perez

**Affiliations:** ^1^Department of Veterinary Population Medicine, University of Minnesota, St. Paul, MN, USA

**Keywords:** FMD, transmission, meta-analysis, simulation model, Delphi technique

## Abstract

In the event of a foot-and-mouth disease (FMD) incursion, response strategies are required to control, contain, and eradicate the pathogen as efficiently as possible. Infectious disease simulation models are widely used tools that mimic disease dispersion in a population and that can be useful in the design and support of prevention and mitigation activities. However, there are often gaps in evidence-based research to supply models with quantities that are necessary to accurately reflect the system of interest. The objective of this study was to quantify values associated with the duration of the stages of FMD infection (latent period, subclinical period, incubation period, and duration of infection), probability of transmission (within-herd and between-herd *via* spatial spread), and diagnosis of a vesicular disease within a herd using a meta-analysis of the peer-reviewed literature and expert opinion. The latent period ranged from 1 to 7 days and incubation period ranged from 1 to 9 days; both were influenced by strain. In contrast, the subclinical period ranged from 0 to 6 days and was influenced by sampling method only. The duration of infection ranged from 1 to 10 days. The probability of spatial spread between an infected and fully susceptible swine farm was estimated as greatest within 5 km of the infected farm, highlighting the importance of possible long-range transmission through the movement of infected animals. Finally, while most swine practitioners are confident in their ability to detect a vesicular disease in an average sized swine herd, a small proportion expect that up to half of the herd would need to show clinical signs before detection *via* passive surveillance would occur. The results of this study will be useful in within- and between-herd simulation models to develop efficient response strategies in the event an FMD in swine populations of disease-free countries or regions.

## Introduction

As the world’s largest beef producer and second largest pork producer, the United States (US) is a major player in the world livestock market (1). The US’s ability to export livestock and livestock products is highly dependent on maintaining a foot-and-mouth disease (FMD)-free status. Although an epidemic has not occurred since the eradication of FMD from the US in 1929, the threat of reintroduction remains due to international travel and trade as seen in recent outbreaks in, for example, the UK, Taiwan, the Netherlands, and France (2, 3). In an effort to contain and control FMD as proficiently as possible, it is common for an affected country to adopt a policy to cease animal movements and depopulate infected animals. However, a strong understanding of FMD spread under regional conditions is essential for efficient preparedness, response, and utilization of resources. Therefore, it is important to carry out analytical studies for strategic and response planning before an outbreak occurs, which may be helped by the formulation, parameterization of, and experimentation with, disease models.

Infectious disease simulation models use mathematics to mimic the dispersion of disease in a population and can be useful in elucidating the mechanisms by which pathogens spread, as well as the underlying processes that influence animal movements, in the geographical region where infection occurs. Stochastic simulation models account for uncertainty and biological fluctuation by using probability distributions to encode for one or more of the variables in the model. However, there are often gaps in evidence-based research to supply models with quantities that are necessary to accurately reflect the system of interest. Researchers and veterinarians with extensive experience may help to fill those gaps and build confidence around the quantity of interest when feasibility restricts the amount of data that can be collected.

The efficacy and speed of FMD virus transmission is dependent on the strain of the virus, the contact structure between hosts, and susceptibility of species involved (4). Therefore, it is critical to develop species-specific transmission values that describe the time course of infection for the host and the probability of transmission. Pigs have played a role in recent outbreaks of FMD. For instance, in the 2011 outbreak in South Korea, the index case occurred on a pig farm where misdiagnosis led to rapid nationwide dissemination, resulting in the ultimate infection of approximately 3,700 farms and the culling of 3.48 million susceptible animals (5).

Here, we quantified parameters associated with FMD transmission in swine using a meta-analysis of the peer-reviewed literature and expert opinion. A modified Delphi technique was applied during a meeting with individuals possessing an average of over 12 years of experience with FMD. In addition, swine practitioners were asked to estimate the proportion of the herd that would need to show clinical signs for the diagnosis of a vesicular disease to occur. Our results will be of use for the parameterization of within- and between-herd FMD transmission models in the US and other FMD-free countries and regions.

## Materials and Methods

### Meta-Analysis

A meta-analysis (6) was conducted to quantify values associated with the time course of FMD infection in swine and was composed of four main components, namely, (1) literature search, (2) inclusion criteria (3) definition of parameter values obtained through the meta-analysis, and (4) statistical analysis including the effects of experimental bias.

#### Literature Search

Literature searches were conducted using two electronic databases, PubMed and Agricola. The searches were conducted using multiple keywords and expressions (“foot-and-mouth disease”[MeSH Terms]) OR (“foot-and-mouth”[All Fields] AND “disease”[All Fields]) OR “foot-and-mouth disease”[All Fields] OR (“foot”[All Fields] AND “mouth”[All Fields] AND “disease”[All Fields]) OR (“foot and mouth disease”[All Fields]) AND (“swine”[MeSH Terms] OR “swine”[All Fields]) AND (“transmission”[Subheading] OR “transmission”[All Fields]) and swine AND foot and mouth disease AND transmission, respectively. Titles and abstracts were imported into RefWorks citation manager for review.

#### Inclusion Criteria

Inclusion criteria for this study include experimental studies that investigate direct and indirect transmission of FMD between unvaccinated domesticated swine with individual-level infection data.

#### Definition of Parameters Estimated through the Meta-Analysis

**Figure 1:**
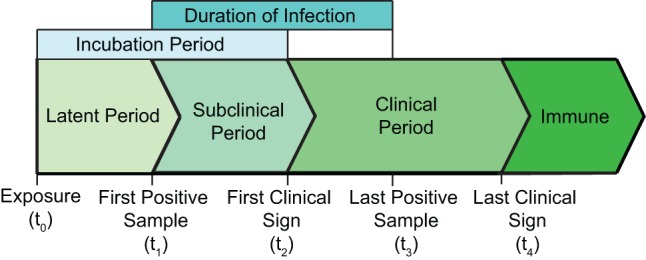
**Time course of FMD infection in pigs infected through contact with an inoculated pig**.

Parameter values associated with the time course of FMD infection were defined as the latent period, subclinical period, incubation period, and duration of infection (Figure 1). The duration of the stages of FMD infection were described as the following: the latent period (*t*_0_–*t*_1_) was considered the time from exposure to the time sample collection resulted in the first positive sample (oral swabs, nasal swabs, or blood); the subclinical period (*t*_1_–*t*_2_) was described as the time from sample collection resulted in the first positive test to the development of clinical signs (increased body temperature, lameness, dullness, reluctance to stand, and presence of vesicular lesions), and the duration of infection (*t*_1_–*t*_3_) was described as the time from sample collection of the first positive test until sample collection of the last positive test result (virus isolation or RT-PCR). The latent period, subclinical period, clinical period, and incubation period were determined from transmission studies using the first positive test and clinical signs in contact pigs. Studies that reported these time periods in hours were converted into days and were rounded to the nearest day. For studies that reported the incubation period and latent period, the subclinical period was calculated by subtracting the duration of the latent period from the incubation period.

#### Statistical Analysis

One parametric survival regression model was fit for each of the stages of infection (latent period, subclinical period, incubation period, and duration of infection) to identify factors that influence the stages of FMD infection extracted from experimental studies. This method is an adaptation of the time-to-event modeling method used by Mardones et al. (7) to estimate the time ratio of an event in an accelerated failure time (AFT) regression model. The AFT model was fitted using the survreg function in the Survival package in R (8). The survival regression model assumed that the baseline hazard function followed a Weibull distribution, which is appropriate for data exhibiting a monotonic hazard rate. The time ratio of the AFT model describes the relative increase in time to the event compared to the baseline. The following factors were fit in the regression: diagnostic test, duration of contact with inoculated pig, reference laboratory, ratio of inoculated seeder pigs to susceptible contact pigs, sampling method, and strain (Table 1). Survival data were fitted and compared through a stepwise approach using the Akaike information criteria (AIC) (9). Factors, covariates, and interactions terms that produce the lowest AIC were calculated using the stepAIC function in the MASS package in R (10, 11) to select the most informative variables. Individual factors that resulted in a statistically significant model (*p* < 0.05) were included in the final model. A frailty term, comparable to a random effect in regression models, was included in the models to adjust for the variability between individual experiments. The frailty term was retained in the final model only if it improved the AIC.

**Table 1:** **Variables considered in the accelerated failure time model**.

Variable	Explanation	Description
Diagnostic test	Test chosen for the detection of FMDV	RT-PCR
		Virus isolation
Duration of contact	Time that infected inoculated pigs and susceptible pigs were housed together	Quantified in days
Reference laboratory	Laboratory at which the experiment was conducted	Lelystad
		Pirbright
		Plum Island
Ratio of inoculated to contact pigs	Number of inoculated pigs/number of susceptible pigs in contact	Quantified as the number of inoculated/the number of susceptible
Sample	Tissue or excreta collected for FMDV identification	Serum
		Nasal swabs
		Oropharyngeal fluid
Strain	Strain of FMDV used to infect inoculated pig	O/TAW/97 O/NET/2001
		O/HKN/21/70
		O/UKG/01
		O/SKR/2000
		O/TAW/0/2/99
		A24 Cruzeiro
		O1 Manisa
		Asia 1 Shamir

**Figure 2:**
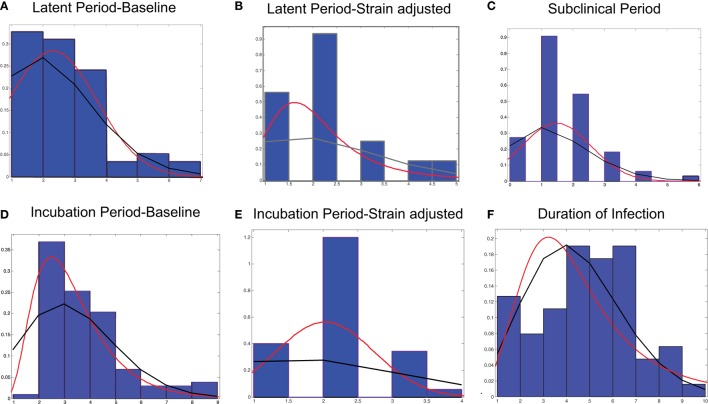
**Frequency distributions and probability distributions for the stages of FMD infection in pigs using data obtained through experimental studies**. **(A)** Latent period-baseline, **(B)** latent period-strain adjusted, **(C)** subclinical period, **(D)** incubation period-baseline, **(E)** incubation period-strain adjusted, and **(F)** duration of infection. The lines represent continuous (red) and discrete (black) probability distributions.

Probability distribution functions were fit by investigating distributions commonly used and those used in FMD simulation models (7, 12, 13) and included: binomial, exponential, Inverse Gaussian, Poisson, Pearson 5, Weibull, Log-logistic, and normal distributions. Continuous and discrete theoretical distributions of the duration of the stages of FMD infection were selected using the Anderson–Darling goodness of fit test for continuous data and the Chi-square test for discrete data using @Risk (14) (Figure 2). Bin size was selected using the Freedman–Diaconis Rule. We then considered the conceptual aspects of the distributions and choose the simplest, most accurate distribution.

### Expert Selection

Five individuals external to the University of Minnesota were selected based on their training and experience with FMD. Expert experience ranged from 12 to 35 years working with FMD, including experts with specialized area of knowledge in academia (*n* = 2), field experience (*n* = 3), government work (*n* = 4), and laboratory experiments (*n* = 3).

### Data Collection

Data were collected utilizing a modification of the Delphi technique, an accepted method of obtaining data on a real world issue (15). Here, we used a two-round approach to reach consensus on transmission data relating to FMD.

#### Round 1

Through an open-ended questionnaire, experts were asked questions about the incubation period, mortality rates (adult pigs and piglets), probability of transmission, and spatial spread (at 1, 5, 10, and 50 km from an infected farm). The questionnaire was created based on extensive literature review, and the questions were the same for all experts.

These data were recorded by the respondents on paper, reviewed, and transferred to electronic format. The questionnaire was used as a survey instrument to collect data in Round 2.

#### Round 2

In the second round, each participant was asked to review the items from the initial questionnaire to discuss the reasoning supporting the response. In the case of incompatible answers, responses were discussed until unanimous understanding and consensus was reached.

### Swine Practitioner Survey

Twenty surveys were administered to swine practitioners attending the 2015 Leman Swine Conference in St. Paul, MN, USA. The survey asked practitioners to estimate the proportion of a swine herd (typical size) that would need to show clinical signs before a vesicular disease was suspected.

### Data Analysis

Survey responses were recorded and distributions were fit for FMD incubation period, disease-associated mortality rate, transmission probability, spatial spread, and proportion of the herd clinical for diagnosis to occur. Questionnaire results were described using the BetaPERT probability distribution function for the minimum, most likely, and maximum values for the mortality rates, probability of transmission, and spatial spread (Table 2). The estimation of mortality is the percentage of the herd that died due to disease. It was estimated separately for adult pigs and piglets.

**Table 2:** **Estimations of disease induced mortality rates and the probability of transmission given direct contact**.

Parameter description	Distribution
Adult mortality (%)	BetaPERT (12.5, 20.8, 40.0)
Piglet mortality rate (%)	BetaPERT (18.3, 58.3, 23.0)
Transmission probability (direct contact %)	BetaPERT (46, 84, 97.5)

*Values were obtained through expert opinion*.

The probability of spatial transmission was defined as the probability that farm *j* becomes infected by farm *i* through a route described in any manner other than through the direct movement of animals. The probability of spatial transmission was estimated at a distance of 1, 5, 10, and 50 km from the infected premises. The expert-solicited most likely probability of spread was plotted at each distance and a non-linear function was fitted to the data in MATLAB using the Curve Fitting App (16).

## Results

### Meta-Analysis

#### Literature Search

The PubMed and Agricola search resulted in 216 and 54 articles, respectively. Literature search results were screened for duplicate articles. Individual titles and abstracts were read to determine if the article met the inclusion criteria. Articles that did not specifically address FMD virus transmission between swine were excluded. After removing duplicate articles and excluding studies that did not meet the inclusion criteria, seven articles remained. The articles were published between 2003 and 2012 and include three serotypes (O, A, and Asia1) and nine strains (Table 3). Experiments were conducted at three reference laboratories, including the Central Institute for Animal Disease Control (CIDC, Lelystad, The Netherlands), the Institute for Animal Health (IAH, Pirbright, UK), and the Plum Island Animal Disease Center (PIADC, New York, NY, USA).

**Table 3:** **Experimental studies used to fit distributions for the latent, subclinical, and infectious period**.

Reference	Reference laboratory	Strain	Number of pigs (contact only)
Alexandersen et al. (17)	Pirbright	O/TAW/97	12 (6)
Eblé et al. (18)	Lelystad	O/TAW/97	50 (25)
Howey et al. (19)	Pirbright	O/UKG/01	12 (0)
Orsel et al. (20)	Lelystad	O/NET/2001	34 (25)
Pacheco and Mason (4)	Plum Island	O/HKN/21/70	42 (18)
		O/TAW/97	
		O/UKG/01	
		O/SKR/2000	
		O/TAW/0/2/99	
Pacheco et al. (21)	Plum Island	A24 Cruzeiro	30 (18)
		O1 Manisa	
		Asia 1 Shamir	
Quan et al. (22)	Pirbright	O/UKG/01	70 (38)
Van Roermund et al. (23)	Lelystad	O/NET/2001	36 (24)

#### Time Course of FMD Infection

##### Latent Period

The experimental studies obtained through the literature review revealed that the latent period ranged from 1 to 7 days. This was in agreement with the experts’ response in which the latent period ranged from 1 to 5 days with the majority of pigs testing positive on day 1 (data not shown). The first stepwise AIC calculation indicated that the latent period was significantly influenced by strain, reference laboratory, and time of introduction and whether the pig was infected through inoculation or contact. While inoculation *via* heel bulb or intravenous injection is a common technique, it may not be a realistic approach to estimate the duration of infectious stages of FMD in a population infected through direct contact. Because inoculated animals are not biologically representative of natural conditions and have a decreased latent period, inoculated pigs were excluded from the analysis. The final model included strain and sampling method, and a frailty term for the individual experiments, suggesting a baseline latent period of 3.63 days (*p* < 0.001) (Table 4). Samples collected through oropharyngeal swabs resulted in shorter latent periods. The latent period was adjusted for strain by separating those with a significantly shorter time ratio (Table 4) and fit to Binomial, Normal, and Log-logistic distributions (Figures 2A,B; Table 5).

**Table 4:** **Accelerated failure time model fitted for the latent period, and incubation period (Weibull distribution, shape parameter latent period = 2.34, shape parameter incubation period = 3.41)**.

Time period	Variable		Time ratio	β	95% CI	*p*-Value
Latent period	Strain	A24 Cruzeiro	0.28	−1.27	(−2.21, 0.32)	<0.01
		O/HKN/70	0.23	−1.47	(−2.45, −0.49)	<0.005
		O/NET/2001	0.31	−1.17	(−2.24, −0.11)	<0.05
		O/TAW/97	0.30	−1.22	(−2.12, −0.32)	<0.01
Incubation period	Strain	O/HKN/70	0.43	−0.85	(−1.31, −0.38)	<0.001
		O/TAW/97	0.59	−0.53	(−0.92, −0.14)	<0.001
		O/SKR/00	0.53	−0.64	(−1.14, −0.15)	0.01

*Baseline latent period = 3.63 days, baseline incubation period = 4.66 days*.

**Table 5:** **Descriptive statistics of (a) discrete and (b) continuous distributions fit to the stages of FMD infection in pigs**.

Stage of infection	Distribution	Parameters
Latent period-baseline (*t*_0_–*t*_1_)	(a) Binomial	(a) *N* = 58, *p* = 0.04
	(b) Normal	(b) μ = 2.31, σ = 1.40
Latent period-adjusted (*t*_0_–*t*_1_)	(a) Binomial	(a) *N* = 97, *p* = 0.02
	(b) Log-logistic	(b) μ = 0.65, σ = 0.28
Subclinical period (*t*_1_–*t*_2_)	(a) Binomial	(a) *N* = 66, *p* = 0.02
	(b) Normal	(b) μ = 1.48, σ = 1.10
Incubation period-baseline (*t*_0_–*t*_2_)	(a) Binomial	(a) *N* = 103, *p* = 0.03
	(b) Inverse Gaussian	(b) μ = 3.36, λ = 16.97
Incubation period-adjusted (*t*_0_–*t*_2_)	(a) Binomial	(a) *N* = 35, *p* = 0.06
	(b) Normal	(b) μ = 2.03, σ = 0.71
Duration of infection (*t*_1_–*t*_3_)	(a) Poisson	(a) λ = 5.19
	(b) Log-logistic	(b) μ = 1.50, σ = 0.40

*Baseline and adjusted values correspond to results of the accelerated failure time model. Definition of parameter values by distribution: binomial – *N* = number of Bernoulli trials, *p* = probability of success; normal – μ = mean, σ = standard deviation; Log-logistic – μ = scale, σ = shape; Inverse Gaussian – μ = mean, λ = shape; and Poisson – λ = mean number of events per interval*.

##### Subclinical Period

A wide range of values, 0–6 days, was estimated for the subclinical period obtained through the experimental studies. The stepwise AIC calculations indicated that inclusion of the route of infection (inoculation vs. contact) in the model produced the best prediction for the subclinical period. Since inoculated animals are not biologically representative of natural conditions and have a decreased subclinical period, inoculated pigs were excluded from the analysis. After excluding animals infected through inoculation, sample method was the only covariate that remained in the best prediction model according to the AIC. However, inclusion of the sampling method produced a non-significant result. The subclinical period was fit to Binomial and Normal distributions (Figure 2C; Table 5).

##### Incubation Period

The incubation period was characterized by values obtained through the literature review, which ranged from 1 to 9 days (Figures 2D,E; Table 5) and was concurrent with the values obtained through expert opinion (min = 2, max = 9) (data not shown). The final model included strain and sampling method and a frailty term for the individual experiments, suggesting a baseline latent period of 4.66 days (*p* < 0.001) (Table 4). The incubation period of the experimental data was adjusted for strain and fit to Binomial and Inverse Gaussian distributions (Figures 2D,E; Table 5).

##### Duration of Infection

The duration of infection ranged from 1 to 10 days and was fit to a Poisson and Log-logistic distribution (Figure 2F; Table 5). The stepwise AIC calculation indicated that inclusion of the reference laboratory produced the model with the lowest AIC, with the baseline duration of infection estimated to be 6.23 days. Inclusion of the reference laboratory and frailty term for the individual experiment in the final model resulted in a statistically significant model (*p* < 0.001).

### Expert Opinion

#### Spatial Transmission

**Figure 3:**
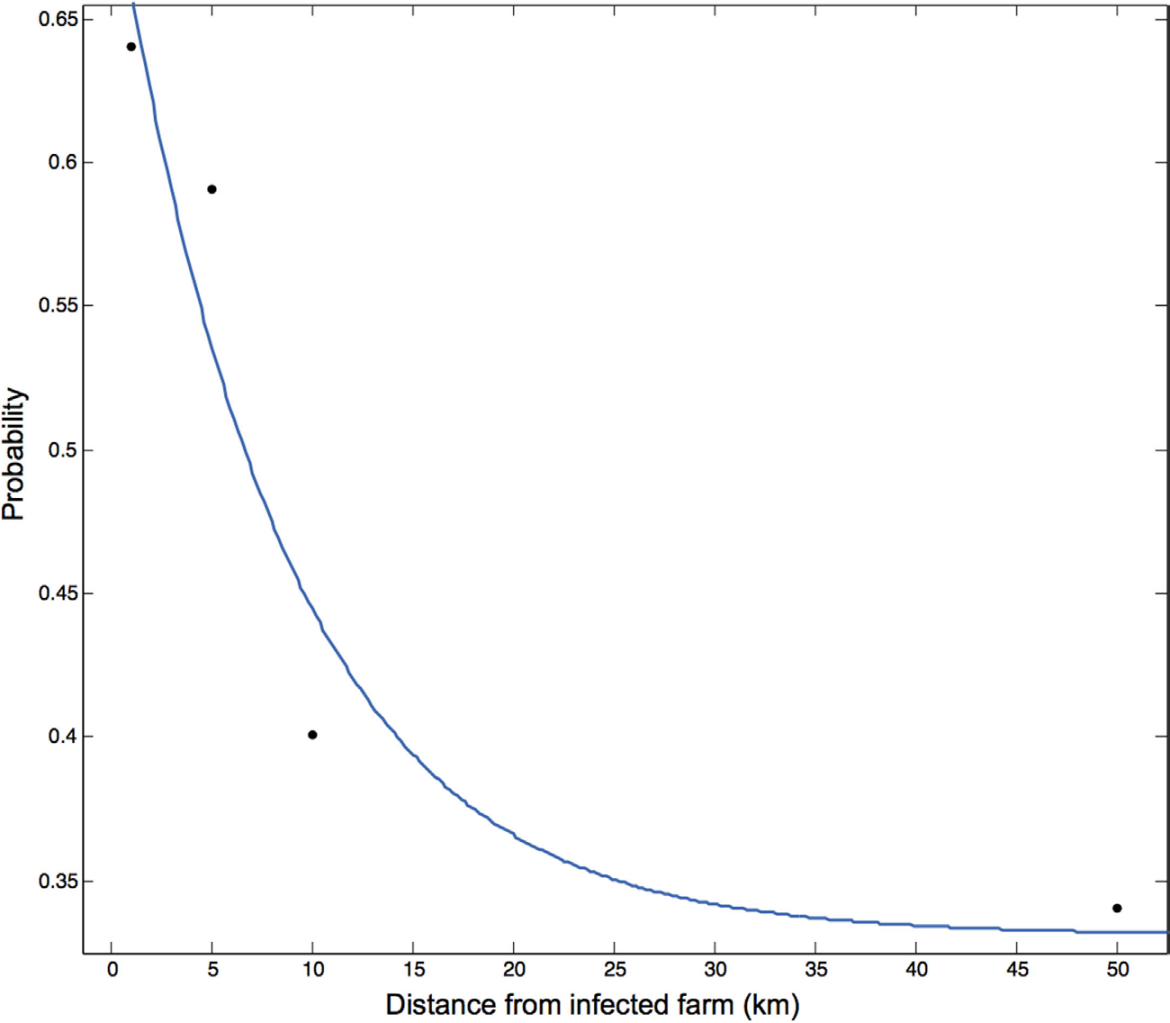
**Transmission kernel calculated from the probability of transmission through indirect contact at 1, 5, 10, and 50 km prior to the implementation of control measures**. The probability of transmission is described by the equation *P*(*x*) = 3.693 × *e*^(−0.118*x*)^ + 0.3307 where *P*(*x*) is the probability of spatial transmission between infected farm *i* and susceptible farm *j* located *x* distance apart in kilometers. Values were obtained through expert opinion.

The probability of transmission from infected farm *i* to susceptible farm *j* was estimated by expert opinion at a distance of 1, 5, 10, and 50 km from the infected premise and was described by the expression *P*(*x*) = *a* × *e*^(−*bx*)^ + *c* where the coefficients and the corresponding 95% CI were *a* = 0.3693 (−1.079, 1.818), *b* = 0.1182 (−0.9134, 1.15), and *c* = 0.3307 (−0.6243, 1.286) (adjusted *R*^2^ = 0.745) (Figure 3).

#### Swine Practitioner Survey

**Figure 4:**
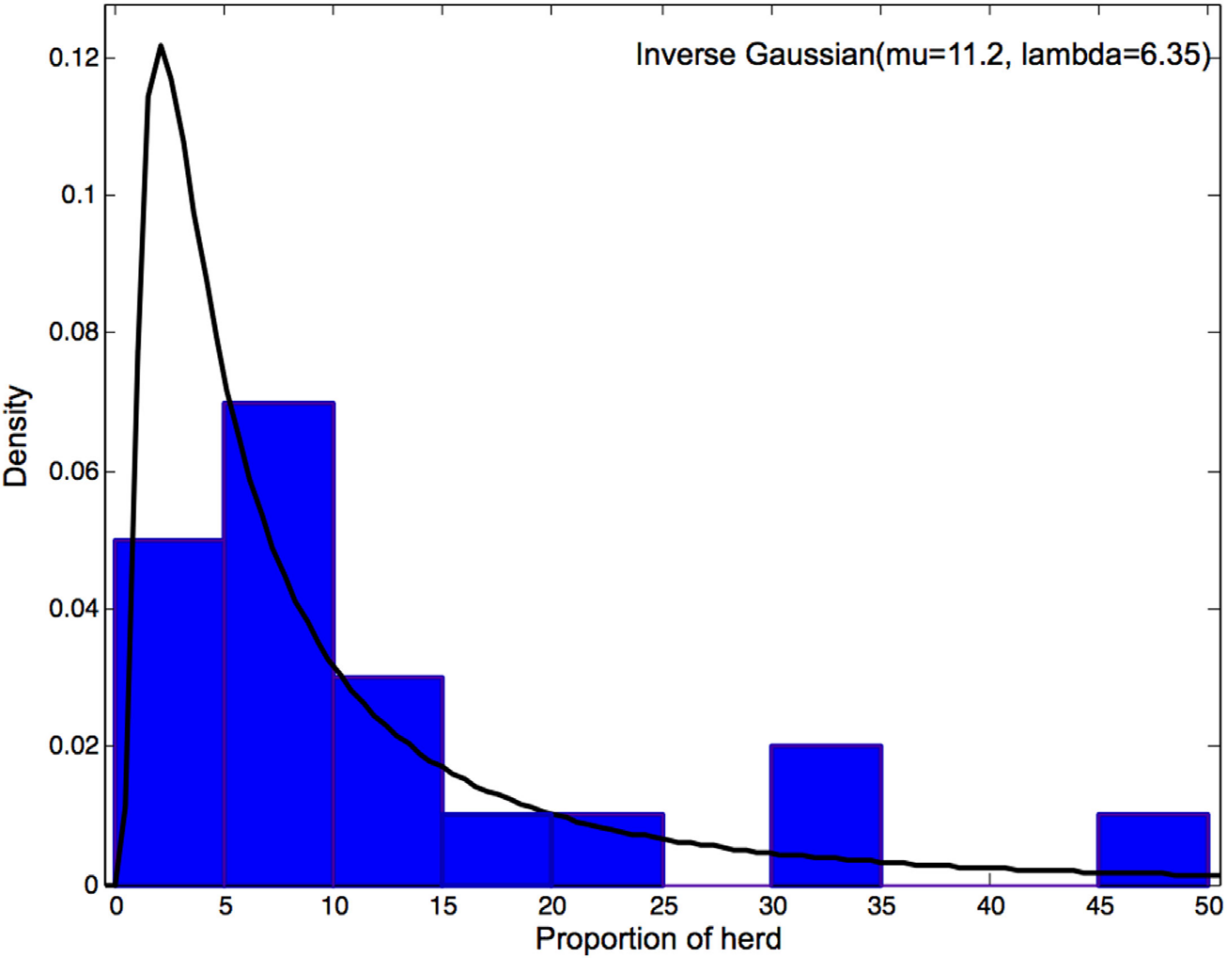
**Distribution of the estimated proportion of the herd showing clinical signs for diagnosis of a vesicular disease to occur**.

Swine practitioners estimated the proportion of the herd that would need to show clinical signs for the diagnosis of a vesicular disease to occur. This estimation ranged from 1 to 50% with a mean of 11.2% and a median of 5.75 (Figure 4). An Inverse Gaussian distribution was the best fit according to the Anderson–Darling goodness of fit test.

## Discussion

To the best of our knowledge, this is the first study aimed to quantify parameters associated with FMD transmission in swine for use in transmission models, using both expert opinion and meta-analyses of published studies. We employed a modified Delphi technique to individuals with at least 12 years of experience with FMD. In addition, we asked swine practitioners to estimate the proportion of the herd that would be affected for the diagnosis of a vesicular disease to occur which can be used to estimate the proportion of the herd that would be subclinical at the time of diagnosis. Results reported here will be valuable for developing simulation models of FMD transmission in swine farms.

Existing models of FMD vary in approach. As a result, the parameter values required for the models also differ. A common approach to quantify parameter values is to use existing disease data. For instance, a recent review of data-driven models of FMD revealed that data from 12 different epidemics have been used in models and that more than half used data from the 2001 UK epidemic (24), where pigs were not largely involved. However, transmission characteristics of FMD infection are influenced by biological processes specific to the strain of FMD virus, host, and environmental factors, such as the rate of contact (17, 25) and variations of parameter values, associated with these factors should be considered.

In a previous study (7), the duration of infection stages of FMD was reported for serotype O. They found that experimental conditions, such as host species involved in the transmission study and specific virus strain, significantly influenced the time course of disease. By utilizing a stepwise regression analysis similar to that described by Mardones et al. (7), we were able to update the parameters distributions with current studies of FMD transmission in swine. Moreover, we were able to provide a range of values that play a key role in between-farm disease transmission models including time to detection and the probability of spatial spread.

The studies included in our analyses aim to understand the determinants of transmission through controlled experiments by varying factors within each experiment and measuring the impact of that factor on the kinetics of viral shedding and the manifestation of clinical disease. The factors measured in the experiments were extracted from the studies and included strain, duration of contact, route of infection, ratio of inoculated to contact pigs, and method of sampling. Of these factors, we found that the latent period and incubation period was shorter in inoculated animals than animals infected through direct contact. While the inoculation of donor animals is essential to reliably produce infectious animals with clinical disease, using the time course of disease in these animals to estimate the latent period is not appropriate as direct inoculation evades the host first line of defenses against infection (21). Current studies of FMD in swine suggest that initial virus entry occurs at the lymphoid tissues of the pharyngeal region followed by low-level viremia, then replication and development of vesicles in epithelial tissues (17, 24). Much greater amplification of the virus occurs in the epithelial cells leading to a substantially greater, detectable level of viremia in the pig (26, 27). It is likely that pigs infected through intradermal heel bulb or intravenous inoculation bypass the initial phase of infection leading to shorter latent periods than pigs infected through contact.

The frequency distribution for the latent period, subclinical period, incubation period, and duration of infection are consistent with those estimated in the Mardones et al. (7) paper. The frequency distributions are right skewed with relatively short tails. But the range of the values obtained in this study was consistently shorter for each of the stages of infection, and the duration of infection was shorter for a greater proportion of individuals represented in this study. This is likely due to the differences in the experimental design of the studies captured in our literature search such as the duration of the experiment and strain of virus. Also, in agreement with the study by Mardones et al. (7), we found that strain and method of sampling significantly influence the latent period and incubation period of FMD infection. These findings suggest that models will benefit from the inclusion of strain-specific factors and that sampling oropharyngeal fluid may be helpful in identifying infected individuals in the early stages of an outbreak or during active surveillance.

For the definitive diagnosis of FMD to occur, clinical disease must be recognized, and the identification of live virus must occur. In an FMD-free country, a producer or veterinarian identifies the lesions in the index case through passive surveillance. Once the index case has been confirmed, and the outbreak is underway, diagnosis may occur solely through clinical signs. While it seems implausible that up to 50% of a herd would be showing clinical signs before clinical disease is recognized, individuals who work with animals on a daily basis may fail to recognize the clinical signs due to inexperience (28–30). For instance, during the 2001 UK State Veterinary Service FMD investigations, veterinary officers found that up to 90% of 527 pigs on the index farm had lesions consistent with FMD (31). Delay in the time to diagnosis in the index case can greatly increase the probability of between-herd transmission likely leading to a larger outbreak. However, these results represent the belief of the limited number of practitioners surveyed in the study here and may not be representative of every swine farm in the country.

An additional caveat is that we used the opinion of experts to quantify parameter values associated with FMD infection. Although the experts in our study had a wide range of experience and extensive amount of time working with FMD, there is such a high degree of uncertainty quantifying values associated with transmission at the population level that error is possible. Between-farm values estimated from this study are useful for parameterizing or model fitting and should be interpreted in light of current research and continually updated for use in disease simulation models. However, for estimating distributions for stages of infection, expert opinion was used as a confirmatory cross-validation of the results of the meta-analysis.

In conclusion, we found that the stages of FMD infection were influenced by route of infection, strain, and sampling method. While modeling efforts may not need to be conducted for every strain of interest, strain variation should be accounted for in the model. Additionally, the probability of spatial spread between an infected and fully susceptible swine farm is greatest within 5 km of the infected farm, highlighting the importance of possible transmission beyond this through the movement of infected animals. Finally, while most swine practitioners are confident in their ability to detect a vesicular disease with few animals showing clinical signs; yet, a small proportion expect that up to half of the herd would need to show clinical signs before detection occurred.

## Author Contributions

Each of the authors were substantial contributors to the conception or design of the work (AK, KV, GP, and AP); or the acquisition (AK, KV, GP, and AP), analysis (AK, GP, KV, and AP), or interpretation of data for the work (AP, KV, GP, AP, and MC), and drafting the work or revising it critically for important intellectual content (AP, KV, GP, AP, and MC). AP, KV, GP, AP, and MC have approved the final version for publication and are in agreement to be accountable for all aspects of the work in ensuring that questions related to the accuracy or integrity of any part of the work are appropriately investigated and resolved.

## Conflict of Interest Statement

The authors declare that the research was conducted in the absence of any commercial or financial relationships that could be construed as a potential conflict of interest. The reviewer [BB] declared a past supervisory role with one of the authors [AP] to the handling Editor, who ensured that the process met the standards of a fair and objective review.

## References

[1] United States Department of Agriculture. Animal Production (03/06/2015) 02/19/2016. Available from: www.usda.gov/wps/portal/usda/usdahome?navid=ANIMAL_PRODUCTION

[2] Yang PC Chu RM Chung WB Sung HT. Epidemiological characteristics and financial costs of the 1997 foot-and-mouth disease epidemic in Taiwan. Vet Rec (1999) 145:731–4. 10972111 10.1136/vr.145.25.731

[3] Garner MG Beckett S. Modelling the spread of foot-and-mouth disease in Australia. Aust Vet J (2005) 83:758–66. 10.1111/j.1751-0813.2005.tb11589.x 16395942

[4] Pacheco JM Mason P. Evaluation of infectivity and transmission of different Asian foot-and-mouth disease viruses in swine. J Vet Sci (2010) 11:133–42. 10.4142/jvs.2010.11.2.133 20458154 PMC2873813

[5] Park J Lee K Ko Y Kim S Lee H Shin Y Control of foot-and-mouth disease during 2010-2011 epidemic, South Korea. Emerg Infect Dis (2013) 19:655–60. 10.3201/eid1904.121320 23632094 PMC3647416

[6] Field AP Gillett R. How to do a meta-analysis. Br J Math Stat Psychol (2010) 63:665–94. 10.1348/000711010X502733 20497626

[7] Mardones F Perez A Sanchez J Alkhamis M Carpenter T. Parameterization of the duration of infection stages of serotype O foot-and-mouth disease virus: an analytical review and meta-analysis with application to simulation models. Vet Res (2010) 41:45. 10.1051/vetres/2010017 20205988 PMC2850150

[8] Therneau T A Package for Survival Analysis in S. R Package Version 2.37-4. Box, 980032:23298–20032 (2013). Available from: http://CRAN.R-project.org/package=survival

[9] Burnham KP Anderson DR Multimodel inference understanding AIC and BIC in model selection. Sociol Methods Res (2004) 33:261–304. 10.1177/0049124104268644

[10] R Core Team. R: A Language and Environment for Statistical Computing. Vienna, Austria: R Foundation for Statistical Computing (2012).

[11] Yamashita T Yamashita K Kamimura R A stepwise AIC method for variable selection in linear regression. Commun Stat Theory Methods (2007) 36:2395–403. 10.1080/03610920701215639

[12] Bates TW Thurmond MC Carpenter TE. Description of an epidemic simulation model for use in evaluating strategies to control an outbreak of foot-and-mouth disease. Am J Vet Res (2003) 64:195–204. 10.2460/ajvr.2003.64.195 12602589

[13] Carpenter TE Thurmond MC Bates TW A simulation model of intraherd transmission of foot and mouth disease with reference to disease spread before and after clinical diagnosis. J Vet Diagn Invest (2004) 16:11–6. 10.1177/104063870401600103 14974841

[14] Palisade Corporation. @RISK. Version 7.0. Ithaca, NY: Palisade Corporation (2016).

[15] Hsu C Sandford BA The Delphi technique: making sense of consensus. Pract Assess Res Eval (2007) 12:1–8. Available online: pareonline.net/getvn.asp?v=12&n=10

[16] The MathWorks, Inc. MATLAB and Statistics Toolbox Release 2012b. Natick, MA: The MathWorks, Inc (2012).

[17] Alexandersen S Quan M Murphy C Knight J Zhang Z. Studies of quantitative parameters of virus excretion and transmission in pigs and cattle experimentally infected with foot-and-mouth disease virus. J Comp Pathol (2003) 129:268–82. 10.1016/S0021-9975(03)00045-8 14554125

[18] Eblé P De Koeijer A Bouma A Stegeman A Dekker A. Quantification of within-and between-pen transmission of foot-and-mouth disease virus in pigs. Vet Res (2006) 37:647–54. 10.1051/vetres:2006026 16777036

[19] Howey R Quan M Savill NJ Matthews L Alexandersen S Woolhouse M. Effect of the initial dose of foot-and-mouth disease virus on the early viral dynamics within pigs. J R Soc Interface (2009) 6:835–47. 10.1098/rsif.2008.0434 19019816 PMC2838353

[20] Orsel K De Jong M Bouma A Stegeman J Dekker A. Foot and mouth disease virus transmission among vaccinated pigs after exposure to virus shedding pigs. Vaccine (2007) 25:6381–91. 10.1016/j.vaccine.2007.06.010 17658199

[21] Pacheco JM Tucker M Hartwig E Bishop E Arzt J Rodriguez LL. Direct contact transmission of three different foot-and-mouth disease virus strains in swine demonstrates important strain-specific differences. Vet J (2012) 193:456–63. 10.1016/j.tvjl.2012.01.012 22342891

[22] Quan M Murphy C Zhang Z Durand S Esteves I Doel C Influence of exposure intensity on the efficiency and speed of transmission of foot-and-mouth disease. J Comp Pathol (2009) 140:225–37. 10.1016/j.jcpa.2008.12.002 19215941

[23] Van Roermund H Eblé P de Jong M Dekker A. No between-pen transmission of foot-and-mouth disease virus in vaccinated pigs. Vaccine (2010) 28:4452–61. 10.1016/j.vaccine.2010.04.019 20416264

[24] Pomeroy L Bansal S Tildesley M Moreno-Torres K Moritz M Xiao N Data-driven models of foot-and-mouth disease dynamics: a review. Transbound Emerg Dis (2015). 10.1111/tbed.12437 PMC5205574 26576514

[25] Quan M Murphy C Zhang Z Alexandersen S. Determinants of early foot-and-mouth disease virus dynamics in pigs. J Comp Pathol (2004) 131:294–307. 10.1016/j.jcpa.2004.05.002 15511538

[26] Alexandersen S Oleksiewicz MB Donaldson AI. The early pathogenesis of foot-and-mouth disease in pigs infected by contact: a quantitative time-course study using TaqMan RT-PCR. J Gen Virol (2001) 82:747–55. 10.1099/0022-1317-82-4-747 11257178

[27] Stenfeldt C Pacheco JM Rodriguez LL Arzt J. Early events in the pathogenesis of foot-and-mouth disease in pigs; identification of oropharyngeal tonsils as sites of primary and sustained viral replication. PLoS One (2014) 9(9):e106859. 10.1371/journal.pone.0106859 25184288 PMC4153717

[28] Bates TW Thurmond MC Hietala SK Venkateswaran KS Wilson TM Colston BW Jr Surveillance for detection of foot-and-mouth disease. J Am Vet Med Assoc (2003) 223:609–14. 10.2460/javma.2003.223.609 12959376

[29] Wee SH Park JY Joo YS Lee JH An SH Control measures implemented during the 2002 foot-and-mouth disease outbreak in the Republic of Korea. Vet Rec (2004) 154:598–600. 10.1136/vr.154.19.598 15162790

[30] McLaws M Ribble C. Description of recent foot and mouth disease outbreaks in nonendemic areas: exploring the relationship between early detection and epidemic size. Can Vet J (2007) 48:1051–62. 17987967 PMC1978293

[31] Gibbens JC Sharpe CE Wilesmith JW Mansley LM Michalopoulou E Ryan JB Descriptive epidemiology of the 2001 foot-and-mouth disease epidemic in Great Britain: the first five months. Vet Rec (2001) 149:729–43. 10.1136/vr.149.24.729 11808655

