# Supplementary material for: Parameter Values for Epidemiological Models of Foot-and-Mouth Disease in Swine
Source: Front Vet Sci. 2016 Jun 1;3:44. doi: 10.3389/fvets.2016.00044 (PMC4887472; doi:10.3389/fvets.2016.00044)
Supplement: Supplementary file 1 [file Data_Sheet_1.DOCX]

**Background and Experience**

1. Which of the following best describes your background? You may select multiple.

a. Laboratory

b. Field

c. Academia

d. Government

2. How many years of experience do you have working with foot and mouth disease in each of these capacities?

3. What state and county do you operate in?

4. Provide example(s) of what you consider to be FMD strains with high / low virulence in swine:

Low virulent:

High virulent:

**Disease Progression**

5. Definition of “Recovered”: Here, we consider recovered animals as those that no longer show clinical signs. They may still be shedding.

| Please use the following table to estimate the progression of disease (% animals affected) following FMDv exposure. For example, if 100 pigs were exposed to FMDv on day 0, what percentage would be shedding by day 2? | | | | | | | | | | | | |  |
| --- | --- | --- | --- | --- | --- | --- | --- | --- | --- | --- | --- | --- | --- |
|  |  |  |  | |  |  |  |  |  |  |  | |  |
| **Number of animals (%):** | **Day 1** | **Day 2** | | **Day 3** | **Day 4** | **Day 5** | **Day 6** | **Day 7 - 10** | **Day 11 - 15** | **Day 15 - 21** | | **Day 22+** |  |
| Shedding Virus (sub-clinical or clinical) |  |  |  | |  |  |  |  |  |  | |  |  |
| Detectable with Laboratory Testing |  |  |  | |  |  |  |  |  |  | |  |  |
| Clinical Signs Present |  |  |  | |  |  |  |  |  |  | |  |  |
| Recovered (no clinical signs) |  |  |  | |  |  |  |  |  |  | |  |  |
| Shedding Quantity* |  |  |  | |  |  |  |  |  |  | |  |  |
| * Please use the following scale: 0-1 where 0 is no shedding and 1 is highest shedding | | | | | | | | | | | | |  |
| How confident are you about the accuracy of these estimates (0-100%)(e.g., I am 95% confident in these estimates)? | | | | | | | | | | | | |  |

**Mortality Rates**

|  |  |  |  |  |  |  |  |
| --- | --- | --- | --- | --- | --- | --- | --- |
| 6. Please estimate the minimum, most likely and maximum *percentage of sows* *and piglets* in an infected herd that die from foot and mouth disease. | | | | | | | |
|  |  |  |  |  |  |  |  |
| **Mortality:** | **HIGH VIRULENCE** | | | **LOW VIRULENCE** | | | **How confident are you about the accuracy of these estimates (0-1)** |
|  | **Minimum Mortality** | **Most Likely Mortality** | **Maximum Mortality:** | **Minimum Mortality** | **Most Likely Mortality** | **Maximum Mortality:** |  |
| Adults (breeding animals) |  |  |  |  |  |  |  |
| Piglet (pre-wean) |  |  |  |  |  |  |  |
|  | | | | | | | |
| COMMENTS: | | | | | | | |

**Transmission Dynamics: Within-herd**

| **7. Transmission Probability** | |  |  |  |  |  |  |
| --- | --- | --- | --- | --- | --- | --- | --- |
|  |  |  |  |  |  |  |  |
| Please estimate the minimum, most likely and maximum probability of transmission through direct contact between one susceptible and one infectious individual for a FMD strain of high and low virulence (sow to sow, sow - piglet, and piglet to piglet- if you think that the probability is the same for all provide only one number). Contact is defined as direct contact (nose-to-nose) between a susceptible and an infectious individual with vesicular lesions. Answers can range from 0-1; 0 = this will *never* happen, 1 = this will *always* happen. | | | | | | | |
|  | | | |  |  |  |  |
| **Transmission Between:** | **HIGH VIRULENCE** | | | **LOW VIRULENCE** | | | **How confident are you about the accuracy of these estimates (0-100%)** |
|  | **Minimum Probability** | **Most Likely Probability** | **Maximum Probability** | **Minimum Probability** | **Most Likely Probability** | **Maximum Probability** |  |
| Sow/Sow |  |  |  |  |  |  |  |
| Sow/Piglet |  |  |  |  |  |  |  |
| Piglet/Piglet |  |  |  |  |  |  |  |
|  |  |  |  |  |  |  |  |
| COMMENTS: | | | | | | | |
|  | | | | | | | |
|  | | | | | | | |
|  |  |  |  |  |  |  |  |

**Transmission Dynamics: Between-herd**

**Spatial spread**

In this section, we aim to capture information about the spread of FMD through mechanisms *other than* direct animal movement (eg. feed, trucks, personnel, aerosol)

| 8. What is the minimum, most likely and maximum probability that a susceptible farm 1, 5, 10, and 50 km away will become infected by a neighboring farm within 1 week? Assume that farms between these do not contribute to FMD transmission. Please quantify how confident you are about the accuracy of your prediction. | | | | | | | |
| --- | --- | --- | --- | --- | --- | --- | --- |
|  |  |  |  |  |  |  |  |
|  |  |  |  |  |  |  |  |
| **Transmission:** | **HIGH VIRULENCE** | | | **LOW VIRULENCE** | | | **How confident are you about the accuracy of these estimates (0-100%)** |
|  | **Minimum Probability** | **Most Likely Probability** | **Maximum Probability:** | **Minimum Probability** | **Most Likely Probability** | **Maximum Probability:** |  |
| Between swine farms: |  |  |  |  |  |  |  |
| 1 km |  |  |  |  |  |  |  |
| 5 km |  |  |  |  |  |  |  |
| 10 km |  |  |  |  |  |  |  |
| 50 km |  |  |  |  |  |  |  |
|  |  |  |  |  |  |  |  |

**Practitioner Questions:**

1. Which of the following best describes your background? You may select multiple.

a. Laboratory

b. Field

c. Academia

d. Government

2. How many years of experience do you have working with foot and mouth disease in each of these capacities?

3. What state and county do you operate in?

4a. Approximately what proportion of a herd would need to show clinical signs (e.g., lameness, off feed, abortions) before a vesicular disease is suspected?

4b. How would this proportion change if it was known that FMD virus was recently diagnosed in the region?
